# Enhanced Solubility, Stability, and Herbicidal Activity of the Herbicide Diuron by Complex Formation with *β*-Cyclodextrin

**DOI:** 10.3390/polym11091396

**Published:** 2019-08-25

**Authors:** Shuang Gao, Jing-Yu Jiang, Yan-Yan Liu, Ying Fu, Li-Xia Zhao, Chun-Yan Li, Fei Ye

**Affiliations:** 1 College of Science, Northeast Agricultural University, Harbin 150030, China; 2 College of Resources and Environment, Northeast Agricultural University, Harbin 150030, China

**Keywords:** diuron, *β*-cyclodextrin, inclusion complex, water solubility, herbicidal activity, thermal stability

## Abstract

The herbicide diuron is hardly soluble in water and most organic solvents and is usually made into a wettable powder or mixed with soil when used, which causes environmental risk and a reduction in herbicidal efficacy. In this study, the physicochemical properties were changed by using *β*-cyclodextrin (*β*-CD) to encapsulate diuron to form an inclusion complex. Some key technologies, including X-ray diffraction (XRD), Fourier transform infrared spectroscopy (FTIR), scanning electron microscopy (SEM), differential scanning calorimetry (DSC), and nuclear magnetic resonance (^1^H NMR), were used to characterize the inclusion complex. The stoichiometry of the inclusion complex was determined by recording the ^1^H NMR spectrum or by using a diagram of inclusion ratios. A phase solubility study proved that the formed inclusion complex exhibited higher water solubility. Thermogravimetric analysis (TGA) demonstrated that the formed inclusion complex exhibited better thermal stability. Biological activity studies indicated that the herbicidal activity, in terms of herbicide removal, of the formed inclusion complex was higher than that of the original diuron. In general, the formation of the inclusion complex could reduce the environmental damage caused by diuron and enhance its herbicidal activity, providing an environmentally friendly method for using diuron.

## 1. Introduction

**Figure 1:**
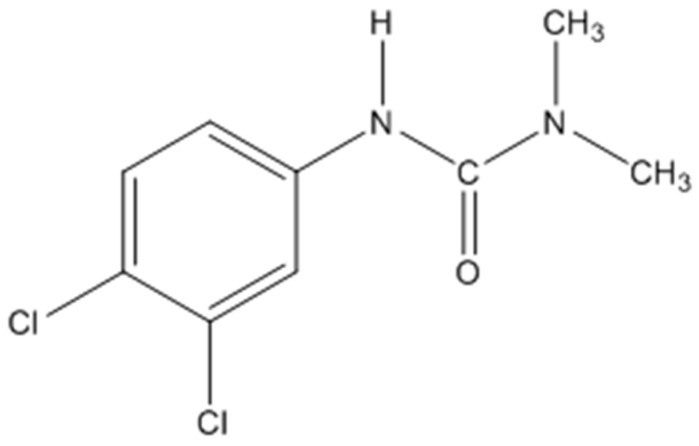
Structure of diuron.

Diuron (Figure 1) is a common systemic herbicide. As a substitute for urea herbicide, it can be absorbed by the roots and leaves of plants, inhibiting the Hill reaction in photosynthesis to control annual and perennial weeds. It is suitable for asparagus, citrus, sugar cane, corn, soybean, peanut, sorghum, cotton, etc. [1]. Due to its poor water solubility, it is usually formulated into a certain proportion of wettable powder or mixed with part of the soil and sprayed. These methods of use are extremely complicated and easily cause residues on crops, resulting in abnormal growth of crops, twisted stems and leaves, no flowers, and no fruit, with the yield of crops being reduced. These residues might even seep into the soil or flow into water bodies, which not only brings harm to the environment, but also threatens human health [2]. According to reports, some crops, such as wheat, suffer from herbicide residues, which cause a yield reduction of more than 35% and even 100% in severe cases. In addition, the residues contribute to drug resistance [3]. Therefore, improving the water solubility and utilization of herbicides is an important issue to be solved. It has been suggested [4] that the formation of an inclusion complex with water soluble host molecules is one of the effective ways to improve the water solubility of herbicides.

**Figure 2:**
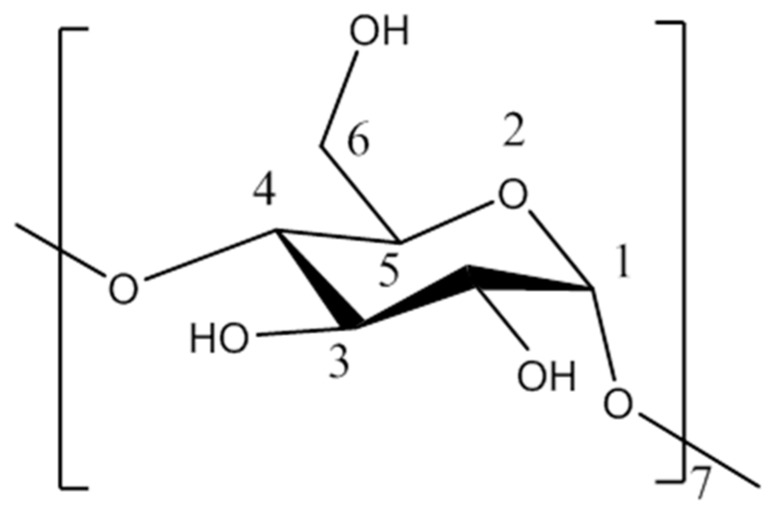
Schematic structure of *β*-cyclodextrin (*β*-CD).

*β*-cyclodextrin (*β*-CD) is a cyclic oligomer composed of seven D-glucopyranoses (Figure 2). It has a hydrophilic outer surface and a hydrophobic inner cavity. *β*-CD can form stable supermolecular complexes with some guest molecules that have matching polarity, size, shape, and properties, or with hydrophobic groups of some guest molecules.

Supramolecular complexes formed by cyclodextrin are widely applied in food, medicine, and agriculture. Its special cone-shaped barrel structure with a cavity is used to encapsulate guest molecules, which can notably improve the solubility, thermal stability, bioavailability, and antioxidant activity of guest molecules [5]. For example, Martina et al. found that α-, *β*-, and γ-CD could be used as generally recognized as safe (GRAS) food additives or soluble fibers, which could control the growth of microorganisms during transportation and preservation, and strongly improve the shelf life of foods [6]. Rubim et al. prepared an amiodarone hydrochloride–*β*-CD (HP*β*CD) inclusion complex by a spray drying method. Compared with the pure drug and physical mixture, the water solubility and drug dissolution rate of the inclusion complex were increased significantly [7]. *β*-CD, as a new drug inclusion material, could not only improve the water solubility and controlled release rate of the drug, but also enhanced the cytotoxicity of the drug in vitro. Lv et al. reported the application of a suspension method for the preparation of an inclusion complex of tetraamine-modified cyclodextrin and rhein, which was a poorly water-soluble drug. It was notable that the significant enhancement in the water solubility and cytotoxicity of rhein in vitro was obtained in the form of a rhein–polyamine–*β*-CD inclusion complex [8]. Pesticides generally have poor water solubility, while cyclodextrin (CD) has been proven to be an effective approach to improve the water solubility of guest molecules by forming inclusion complexes. Therefore, we attempted to use CD to encapsulate pesticides to improve the water solubility and the herbicidal, bactericidal, and insecticidal abilities of pesticides, as well as reduce the environmental pollution caused by the use of pesticides. Gao et al. prepared an inclusion complex of HP*β*CD and fluroxypyr by the saturated aqueous solution method. Results showed that the water solubility, thermal stability, and herbicidal activity of the inclusion complex were improved [9]. Moreira et al. synthesized hydrophobic nanoprecipitates of inclusion complexes formed with *β*-CD and two avermectins named eprinomectin (EPRI) and ivermectin. Experiments showed that *β*-CD improved the thermal stability of avermectins. The EPRI–*β*-CD inclusion complex increased the larvicidal activity against *Aedes aegypti* and reduced the human cytotoxicity of EPRI, which brought better insecticidal effect [10]. Studies have shown that the complexation of butachlor with HP*β*CD reduced the adsorption capacity of soil and enhanced the mobility of butachlor in soil. The dissolution of butachlor in the inclusion complex was significantly increased, and the inclusion complex showed better herbicidal activity compared with free butachlor [11]. The above literature indicated that cyclodextrin inclusion complexes could improve the water solubility, stability, and bioavailability of drug molecules.

In this study, a diuron–*β*-CD inclusion complex was prepared by a saturated aqueous solution [12]. Scanning electron microscopy (SEM), X-ray diffraction (XRD), Fourier transform infrared spectroscopy (FTIR), thermogravimetric analysis (TGA), differential scanning calorimetry (DSC), nuclear magnetic resonance (^1^H NMR), and a phase solubility diagram were used to characterize the physical and chemical properties of *β*-CD, diuron, the diuron/*β*-CD physical mixture, and the diuron–*β*-CD inclusion complex. At the same time, the biological activities of diuron and the diuron–*β*-CD inclusion complex were investigated to compare the herbicidal activities of the inclusion complex and the original diuron. To the best of our knowledge, it was the first investigation of the inclusion behavior of a diuron and *β*-CD complex.

## 2. Materials and Methods

### 2.1. Materials

*β*-CD (purity of 98.00%) and potassium bromide were obtained from Tianjin Guangfu Fine Chemical Research Institute (Tianjin, China). Diuron (purity of 99%) was provided by Shanghai Aladdin Bio-Chem Technology Co., Ltd. (Shanghai, China). Acetone was provided by Tianjin Kermel Chemical Reagent Co., Ltd. (Tianjin, China). Anhydrous ethanol was obtained from Tianjin Zhiyuan Chemical Reagent Co., Ltd. (Tianjin, China).

### 2.2. Preparation Method of the Inclusion Complex

The inclusion complex of diuron and *β*-CD was prepared by co-precipitation, and their molar ratio was controlled to be 1:1. Then, 0.01 mol of *β*-CD was dissolved in 10 mL of deionized water at room temperature to prepare a saturated aqueous solution, and then stirred on a stirrer. Diuron solution was prepared by dissolving 0.01 mol of diuron in 10 mL of acetone. The two solutions were mixed and continuously stirred at 60 °C for 2.5 h. After cooling at 0 °C for 12 h, the suspension was filtered, and the precipitate was washed with acetone to remove residual diuron. Then, the precipitate was stored at 55 °C for 9 h to remove the solvent.

### 2.3. Preparation of the Physical Mixture

The physical mixture was prepared by mixing *β*-CD and diuron in a molar ratio of 1:1, which was the same as the molar ratio when preparing the inclusion complex. The diuron and *β*-CD were thoroughly ground for 15 min to ensure uniform mixing, and then the ground mixture was placed in a 202-2A electrothermal constant temperature drying oven (Tianjin Taisite Instrument Company, Tianjin, China) and dried at a constant room temperature.

### 2.4. Characterization Method

#### 2.4.1. FTIR Analysis

During analysis, appropriate amounts of *β*-CD, diuron, the diuron/*β*-CD physical mixture, and the diuron–*β*-CD inclusion complex were pressed into tablets with KBr, respectively, and the infrared spectrum of each sample was obtained by an ALPHA-T infrared spectrometer (German Brooke Company, Karlsruhe, Germany) at room temperature, with the background of KBr. Baseline correction was applied. The wave number range was 400–4000 cm^−1^.

#### 2.4.2. XRD Analysis

The sample was placed on a test rack using Cu–ĸα (*λ* = 1.5406) rays, 40 kV of tube pressure, and 30 mA of tube flow. At a scanning rate of 2 min^−1^, under the condition of a diffraction angle of 5–90°, the X-ray powder diffraction pattern was finally obtained on a Phillips X-ray diffractometer (Malvern Panalytic, Etten Leur, Netherlands).

The relationship between the spatial orientation of the diffraction line and the crystal structure can be expressed by the Bragg equation (Formula (1)):

2*d*sin*θ* = *nλ* (1)

where *λ* is the wavelength of the X-ray; *θ* is the diffraction angle; *d* is the crystal plane spacing; and *n* is an integer. The wavelength *λ* was measured by a known X-ray diffraction angle and the interplanar spacing, that is, the regular arrangement of atoms or ions in the crystal was obtained.

#### 2.4.3. SEM Study

An SU-8010 environmental scanning electron microscope system (Hitachi Company, Tokyo, Japan) was used to present the state of each sample. The sample was placed on a sample holder with aluminum strips prior to scanning, and the thin gold layer was sputter coated with a 12.5-kV accelerating voltage under high vacuum.

#### 2.4.4. Phase Solubility Study

Excess diuron was added to 10 mL of *β*-CD aqueous solution (0, 0.05, 0.10, 0.50, 1.00, 2.00, 3.00, 4.00, 5.00, 6.00, 7.00, 8.00, 9.00, and 10.00 mmol·L^−1^) in a test tube with stopper. After being shaken at 32 °C for 48 h in a water bath, the mixture was filtered using a 0.45-μm fiber filter, and the obtained filtrate was collected as a sample solution. The amount of diuron drug in each filtrate was measured using a UV–visible dual beam spectrophotometer (UV-2550, Shimadzu, Suzhou, China) at 349 nm.

The phase solubility diagram was plotted to indicate the apparent water solubility of diuron as a function of *β*-CD concentration.

Formula (2) was used to calculate the associated constant value (*K*) [13]:

(2) $$K=\frac{k}{b\left(1-k\right)}$$

where *b* is the solubility of diuron in water, and *k* represents the slope of the phase solubility diagram.

Formula (3) was used to calculate the complexation efficiency (*CE*) [13]:

(3) $$CE=b\times K=\frac{\left[\beta \mathrm{CD}/\mathrm{diuron}\right]}{\left[\beta CD\right]}=\frac{k}{1-k}$$

where [*β*-CD/diuron] refers to the concentration of the inclusion complex, and [*β*-CD] is the concentration of free *β*-CD.

#### 2.4.5. Determination of the Inclusion Ratio

The inclusion ratio was defined as the molar ratio of diuron in the inclusion complex to the total number of diuron and *β*-CD in the inclusion complex. In order to determine the stoichiometry and stability constant, the molar ratio of *β*-CD to diuron was continuously changed while maintaining the total concentration of (c (diuron) + c (*β*-CD) = 2 × 10^−5^ mol·L^−1^). The difference in the absorbances of the mixed solution and the diuron solution at 349 nm (Δ*A*) was recorded under the corresponding conditions. The inclusion ratio of the sample was obtained by plotting Δ*A* versus *r* [14]. The *r* value was calculated using Formula (4):

(4) $$r=\frac{n\left(\mathrm{diuron}\right)}{n\left(\mathrm{diuron}\right)+n\left(\beta \mathrm{CD}\right)}.$$

#### 2.4.6. TGA Study

The *β*-CD, diuron, diuron/*β*-CD physical mixture, and diuron–*β*-CD inclusion complex were used for the TGA test in the thermogravimetric analyzer (Netzsch Company, Shanghai, China), in a constant dry nitrogen gas stream with a scan range of 50–800 °C, at a constant heating rate of 10 °C·min^−1^.

#### 2.4.7. DSC Study

A Perkin–Elmer DSC model 7 was used for recording DSC thermograms of the diuron, *β*-CD, diuron/*β*-CD physical mixture, and diuron–*β*-CD inclusion complex. Samples (3–5 mg) were accurately weighed using a Sartorius 4503 electronic microbalance and heated in closed aluminum crimped cells at a rate of 10 °C·min^−^^1^, at a temperature range between 30 and 200 °C, under a nitrogen flow of 20 mL·min^−1^.

#### 2.4.8. ^1^H NMR Study

^1^H NMR spectra were recorded at 300 MHz with a Bruker AVANCE 300 MHz (BRUKER Inc., Beijing, China). DMSO-*d_6_* (Energy Chemical., Shanghai, China) was used as a solvent, and Tetramethylsilane (TMS) (Energy Chemical., Shanghai, China) was used as an internal standard. The scanning range of the hydrogen spectrum was from –1 to 14 ppm. The ^1^H NMR spectra were processed and analyzed using MestReNova software [15,16].

#### 2.4.9. Detection of Biological Activity

*Echinochloa crusgalli* was chosen as the indicating weed. The seeds were soaked in water at 30 °C for 30 min, and then placed in a petri dish in an incubator at a constant temperature of 28 °C and a humidity of 78% for 48 h to ensure good seed germination.

The soil was sieved with a 2-mm sieve and placed in 20 plastic bowls of 7 × 7 cm in diameter. Fifteen seedlings of the same bud length were sown in each bowl. A layer of soil was gently laid into each bowl to ensure the seeds were completely covered. Finally, each bowl was watered. The plastic bowls were placed in an incubator for 16 days at a constant temperature and divided into five groups, with four bowls in each group. *β*-CD (0.048 mmol/m^2^), diuron (0.048 mmol/m^2^), the physical mixture (0.048 mmol/m^2^ of diuron and 0.048 mmol/m^2^ of *β*-CD), and the inclusion complex (0.048 mmol/m^2^) were sprayed onto the leaves of each group in a 1-m^2^ area, respectively. For the control group, water was sprayed in a bowl with the same volume as the *β*-CD solution. The growth index of *Echinochloa crusgalli* seedlings, including root length, plant height, and fresh weight, was measured after 10 days of incubation in the incubator [17,18]. The measurement procedure was repeated three times.

Chlorophyll content in the *Echinochloa crusgalli* was measured by a spectrophotometer [19]. Then, 0.1 g of the freshly cut sample was weighed by an analytical balance (AUY120 Shimadzu, Japan) and ground with a small amount of 80% acetone, calcium carbonate, and quartz sand. Then, the homogenate was transferred into a centrifuge tube with 80% acetone, while the mortar was washed with 80% acetone to ensure all the homogenate was transferred. The mixture was diluted to 10 mL with 80% acetone and centrifuged at 3000 rpm for 10 min (operating in the dark at room temperature). The supernatant was extracted, and the absorbance of the supernatant was measured at 645 nm and 663 nm to calculate the contents of chlorophyll a and chlorophyll b, respectively, using 80% acetone as a blank. All measurements were repeated three times. The chlorophyll content was derived using Formulas (5) and (6):

*C*_a_ = (12.72*A*_663_ − 2.59*A*_645_)*V*/*W* (5)

*C*_b_ = (22.88*A*_645_ − 4.68*A*_663_)*V*/*W* (6)

where *C*_a_ is the content of chlorophyll a (mg); *C*_b_ is the content of chlorophyll b (mg); *A*_645_ and *A*_663_ represent absorbance at 645 nm and 663 nm, respectively; *V* is a constant volume (mL); and *W* is the nominal sample volume (g).

## 3. Results

### 3.1. FTIR Analysis

**Figure 3:**
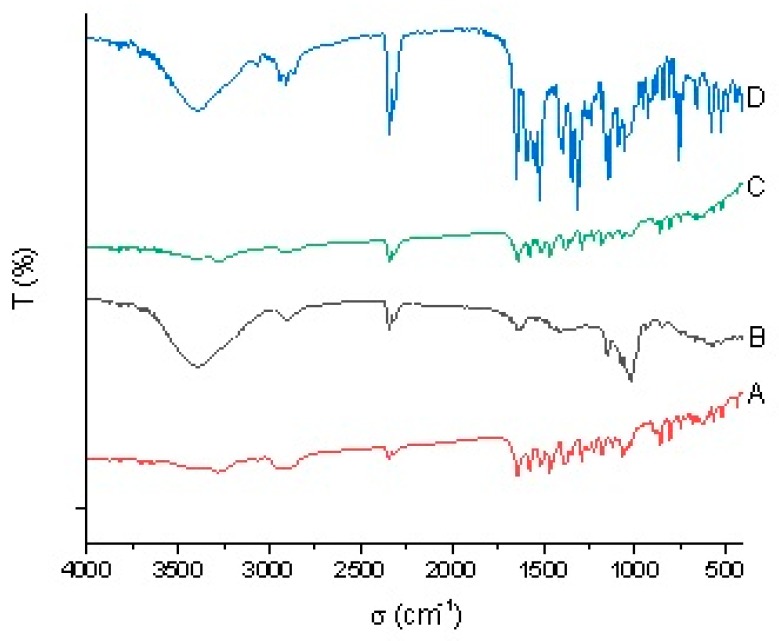
Fourier transform infrared spectroscopy (FTIR) results: (**A**) diuron; (**B**) *β*-CD; (**C**) the inclusion complex; and (**D**) physical mixture.

FTIR analysis was carried out to determine the vibration and angle change between atoms in the molecule as well as information about molecular functional groups of the compound. It was applied to determine the molecular structure and to enable the analysis of mixtures. The chemical structures of unknown substances could be deduced according to the position, shape of absorption peaks, and strength of the absorption bands of different samples. Detailed information is shown in Figure 3.

FTIR spectroscopy provided information about functional groups. From the information provided by FTIR about diuron (Figure 3A), it could be concluded that the peaks at 3243 cm^−1^ and 1620 cm^−1^ were the vibration absorption peaks of N–H and C=O, respectively. The spectrum of *β*-CD (Figure 3B) showed that the specified peaks were at 3409, 2909, 1143, and 1015 cm^−1^. The broad and strong band at 3409 cm^−1^ exhibited the stretching vibration mode of the O–H bond, and the bands at 2909 cm^−1^ corresponded to C–H symmetry in the stretching mode. Furthermore, peaks at 1143 cm^−1^ and 1015 cm^−1^ depicted the presence of C–C and C–O bands, respectively. The FTIR spectrum of the physical mixture of diuron was a simple superposition of diuron and *β*-CD. The diagram of the diuron–*β*-CD inclusion complex was similar to that of *β*-CD, and the N–H and C=O characteristic peaks of diuron completely disappeared, indicating that diuron completely entered the hydrophobic cavity of *β*-CD, which caused changes in the peak position and intensity [20]. FTIR spectrum analysis was one of the favorable types of evidence for explaining the formation of the diuron–*β*-CD inclusion complex [21].

### 3.2. XRD Analysis

**Figure 4:**
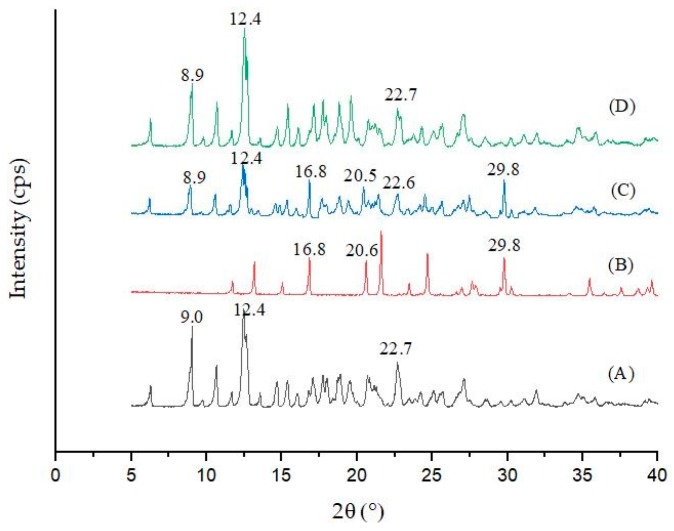
X-ray diffraction (XRD) results: (**A**) *β*-CD; (**B**) diuron; (**C**) physical mixture; and (**D**) the inclusion complex.

XRD analysis refers to the diffraction effect of X-rays in crystal substances for material structure analysis, qualitative analysis of compounds through peak positions, and quantitative analysis of compounds through peak intensities, so as to analyze information such as the structure or morphology of atoms or molecules in substances. XRD can be used to prove whether inclusion compounds are generated [22]. Figure 4 shows the powder X-ray diffraction patterns of *β*-CD, diuron, the diuron/*β*-CD physical mixture, and the diuron–*β*-CD inclusion complex. The main characteristic diffraction peaks of β-CD were at 9.0°, 12.4°, and 22.7° (2θ), and the main characteristic diffraction peaks of diuron were at 16.8°, 20.6°, and 29.8° (2θ). The main characteristic peaks of the powder diffraction pattern of the physical mixture were at 8.9°, 12.4°, 16.8°, 20.5°, 22.6°, and 29.8° (2θ), which was basically a simple superposition of the *β*-CD and diuron diffraction patterns, indicating that that the inclusion complex could not be formed by simple physical mixing of the two substances. The main characteristic diffraction peaks of the inclusion complex were at 8.9°, 12.4°, and 22.7° (2θ), which were very similar to the main characteristic diffraction peaks of β-CD. The characteristic diffraction peaks of diuron had almost disappeared in the inclusion complex, which might be due to the formation of inclusion complexes by diuron in the *β*-CD cavity, and which could be mutually corroborated with the SEM image [23]. From the results of the XRD patterns, strong evidence for the solid inclusion complex of diuron with *β*-CD was provided [24].

### 3.3. SEM Analysis

**Figure 5:**
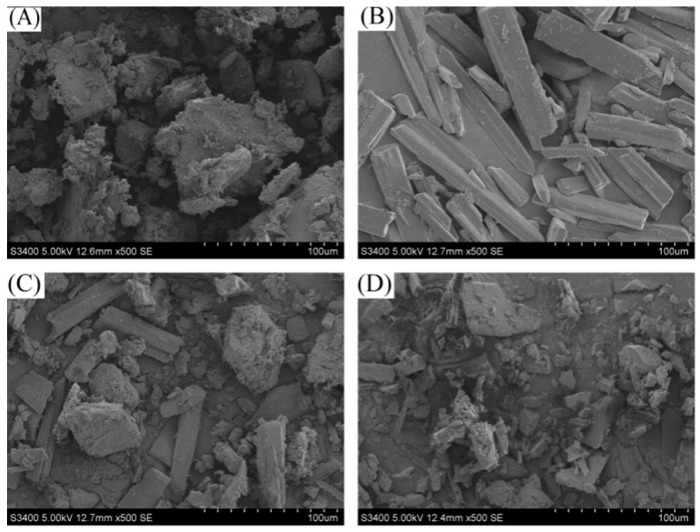
Scanning electron microscopy (SEM) images of (**A**) *β*-CD; (**B**) diuron; (**C**) the physical mixture; and (**D**) the inclusion complex.

SEM, which can directly show the surface morphology of a sample, was used to analyze the surface characteristics of the samples. SEM images of *β*-CD, diuron, the diuron/*β*-CD physical mixture, and the diuron–*β*-CD inclusion complex, which are shown in Figure 5, were compared to determine whether the inclusion compound was formed [25].

As seen in Figure 5, *β*-CD had a prismatic shape and a smooth surface, and diuron presented as a long strip, while the diuron/*β*-CD physical mixture was a simple mixed accumulation of *β*-CD and diuron. The diuron–*β*-CD inclusion complex SEM image was somewhat similar to that of *β*-CD. However, an irregular crystal structure, in which the surface of the substance was somewhat attached, was displayed on the image of the inclusion complex. It can be concluded that the inclusion complex sample was obviously distinct from the other three samples. The reason for this phenomenon is likely related to the formation of the inclusion complex by diuron in the *β*-CD cavity [26].

### 3.4. Phase Solubility Study

As the solubility of insoluble drugs increased after inclusion, the solubility of drugs in cyclodextrin solutions with different concentrations was measured; thus, the solubility of the inclusion compound was obtained, and the stability constant for forming the inclusion compound was calculated [27].

**Figure 6:**
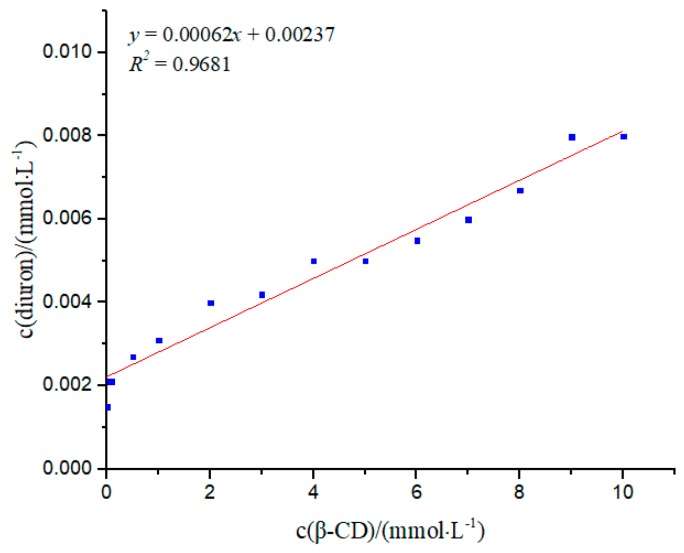
Phase solubility study of the inclusion complex.

According to Inoue et al. [28], the solubility diagram of diuron in *β*-CD (Figure 6) showed solubility changes for diuron in different concentrations of *β*-CD. With the increase in *β*-CD concentration, the concentration of diuron also increased gradually, indicating a clear linear relationship. The solubility diagram of the phase was A_L_-type. A_L_-type inclusions indicate a guest molecule and host molecule interaction with a molar ratio of 1:1. Using Formula (2), the *K* value was determined to be 269.73 mol·L^−1^. When the concentration of *β*-CD was increased to the maximum concentration of 10 mol·L^−1^ during the experiment, the solubility of diuron became 5.3 times the original. The results showed that *β*-CD had a better solubilizing effect on diuron. The *CE* value was calculated according to Formula (3) to be 6.2 × 10^−4^, indicating that *β*-CD had the potential to dissolve diuron molecules.

### 3.5. Inclusion Ratio Study

**Figure 7:**
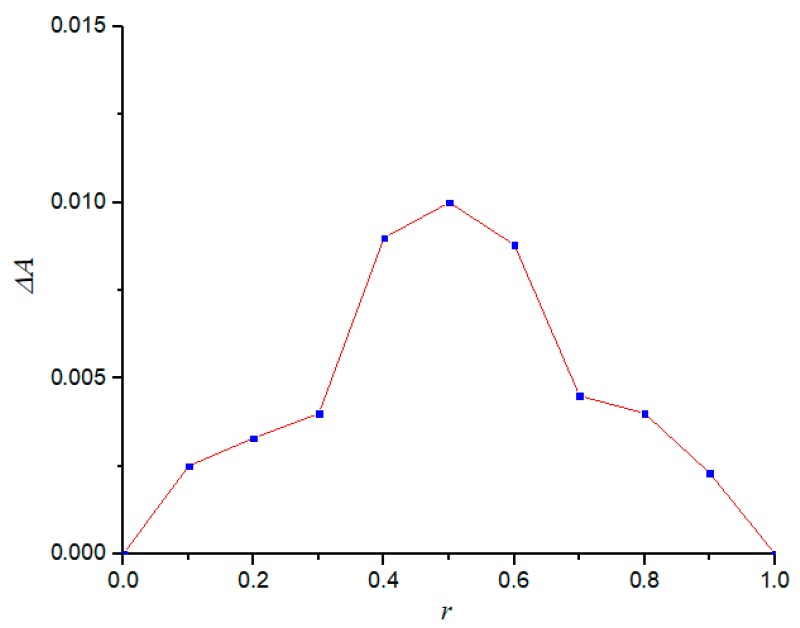
Inclusion ratio study.

Figure 7 shows the measurement results of the inclusion ratio of the diuron–*β*-CD inclusion complex. With the increase of *r*, Δ*A* increased first and then decreased. When *r* was 0.5, the maximum Δ*A* was obtained, which could be used to indicate when the *n* (*β*-CD):*n* (diuron) ratio was 1:1. *β*-CD had the strongest influence on the absorbance of diuron, and could further be used to explain that the inclusion ratio of the diuron–*β*-CD inclusion complex was 1:1 [29].

### 3.6. TGA Study

**Figure 8:**
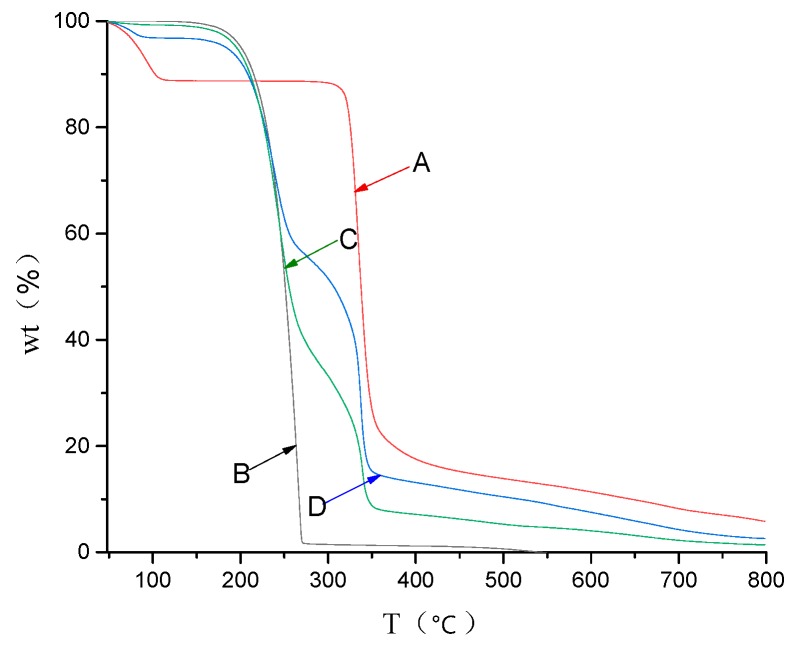
Thermogravimetric analysis (TGA) results: (**A**) *β*-CD; (**B**) diuron; (**C**) physical mixture; and (**D**) the inclusion complex.

The thermal stability of diuron in diuron/*β*-CD was investigated by TGA (Figure 8). TGA provided some reasonable information about guest interaction with CD in a solid state [30]. Pure *β*-CD lost weight in two different temperature ranges. The first weight loss below 110 °C was due to water evaporation, and the second weight loss between 306 °C and 374 °C was a major thermal degradation of *β*-CD. Diuron began to lose weight at 165 °C, which was the highest weight loss ratio. The thermogram of the physical mixture was similar to that of the inclusion complex. However, the inclusion complex decomposed less than the physical mixture in the same temperature range, which exhibited good thermal stability. At the end of the TGA heating scan, diuron, the physical mixture, and the inclusion complex remained at 2.6 wt%, 9.1 wt% and 16.1 wt%, respectively, which indicated that the thermal stability of the inclusion complex was higher than that of diuron. The above results proved that the strong combination of the guest with the *β*-CD cavity could improve its thermal stability [31,32].

### 3.7. DSC Results

**Figure 9:**
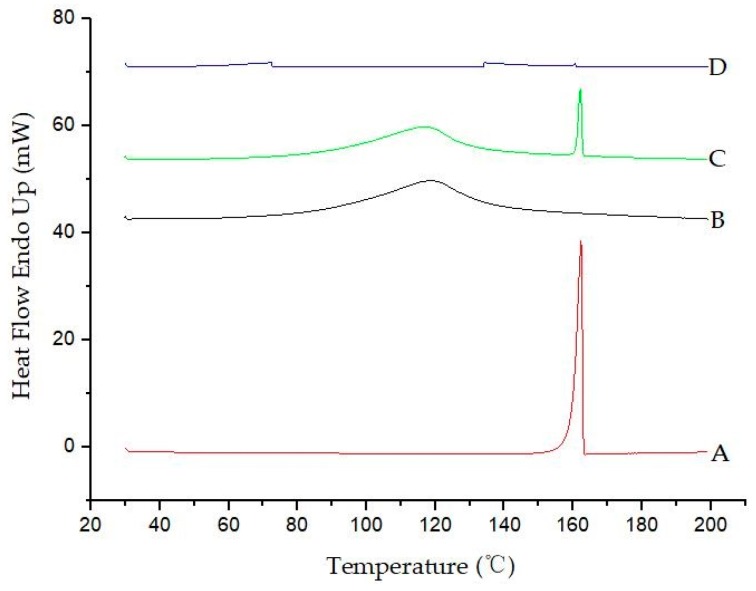
Differential scanning calorimetry (DSC) results: (**A**) diuron; (**B**) *β*-CD; (**C**) physical mixture; and (**D**) the inclusion complex.

DSC technology was used to determine whether the inclusion complex was formed and whether the obtained inclusion complex contained additional free diuron guest molecules that were unincluded [33]. Figure 9 shows the DSC scans of diuron, β-CD, the diuron/β-CD physical mixture, and the diuron–β-CD inclusion complex. When diuron is complexed, it is surrounded by cyclodextrin channel walls to prevent interaction with other diurons [34]. As seen in Figure 9A, the melting peak of diuron was observed at about 162 °C. Liberation of crystal water from β-CD was observed as a broad endothermal peak at around 118 °C. The DSC scan of the physical mixture was a simple overlapping of diuron and β-CD. For the inclusion complex, the melting peaks of diuron and β-CD disappeared completely. This could be considered as indicative of inclusion complex formation and as suggesting that it contained no free diuron [35].

### 3.8. ^1^H NMR Analysis

^1^H NMR was one of the most powerful methods for indicating the formation of a cyclodextrin inclusion complex and calculating stoichiometry [36]. H-3 and H-5, constituting each glucosamine unit of the *β*-CD molecule, were located in the cavity and constituted an inner wall. When the guest molecule entered the *β*-CD cavity, the chemical shift of H-3 and H-5 in *β*-CD was induced to change. Therefore, H-3 and H-5 could be used as spectral probes to study the existence of guest molecules and the interaction between host and guest molecules.

**Figure 10:**
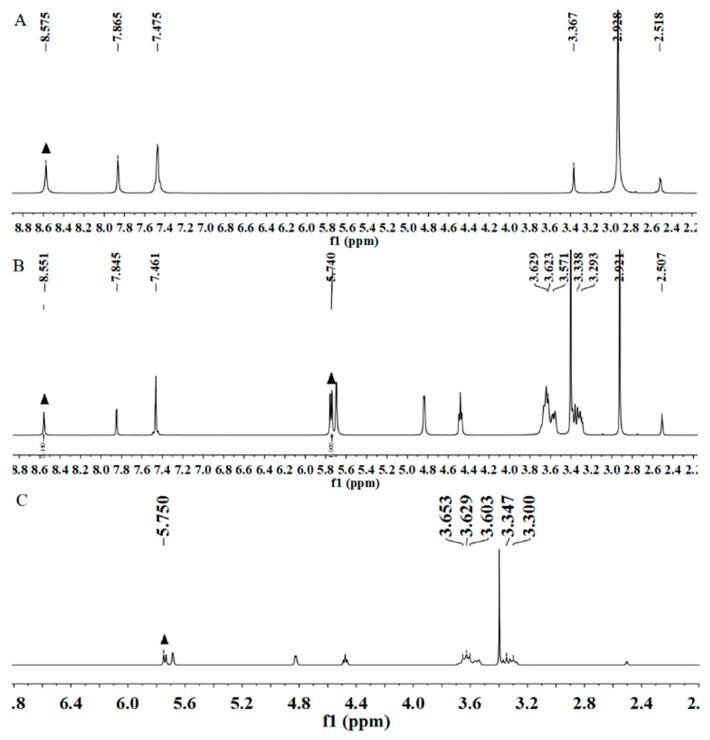
^1^H nuclear magnetic resonance (NMR) spectra: (**A**) diuron; (**B**) the inclusion complex; and (**C**) *β*-CD.

We characterized the formation of inclusion complexes by diuron, the diuron–*β*-CD inclusion complex, and *β*-CD using ^1^H NMR (Figure 10). Table 1 summarizes the chemical shifts of diuron and *β*-CD in the ^1^H NMR spectrum after the formation of the diuron–*β*-CD inclusion complex. It can be seen that the chemical shifts of H-3 and H-5 in *β*-CD changed significantly. The chemical shifts all migrated to the high field. This phenomenon might be caused by the presence of H-3 and H-5 in the *β*-CD cavity. When the inclusion complex was formed, the chemical shift values of H-3 and H-5 varied greatly, while the chemical shift values of H-2, H-4, and H-6 outside the chamber were small. The protons in the *β*-CD cavity moved from various degrees to a high degree, and the peak shape changed significantly, indicating the formation of the diuron and *β*-CD inclusion complex.

The complexation stoichiometric ratio could be obtained by comparing the integral area under the H1 proton for the *β*-CD with that of the H1 protons from the diuron in the inclusion complex spectrum (Figure 10B) [37]. Peaks corresponding to protons of diuron and *β*-CD (Figure 10A,C) were initially assigned. The intensity of the *β*-CD peak was arbitrarily assigned to 1, and the program automatically calculated the strength of the diuron proton. Therefore, the inclusion complex stoichiometry was obtained as follows [38]:

*β*-CD intensity = 1.00 (seven protons)

Diuron intensity = 0.14 (one protons)

Ratio of intensities = (0.14/1)/(1/7) = 0.98

Therefore, the stoichiometric ratio obtained from the ^1^ H NMR data was about 1, which could be mutually corroborated with the diagram of the inclusion ratio.

### 3.9. Results of the Biological Activity Assay

**Figure 11:**
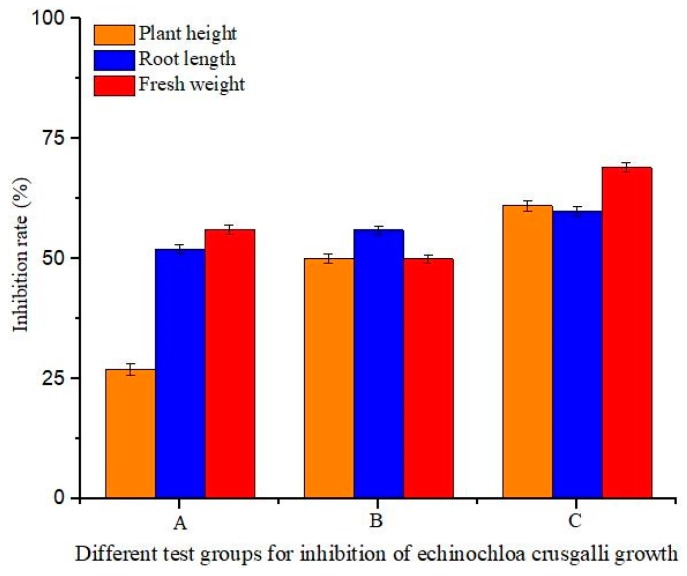
Result of the biological activity assay: (**A**) diuron; (**B**) physical mixture; and (**C**) the inclusion complex.

By comparing the effects of *β*-CD, diuron, the diuron/*β*-CD physical mixture, and the diuron–*β*-CD inclusion complex on the growth of *Echinochloa crusgalli*, their herbicidal activity against weeds was explored. Water was used for the control. The results showed that the plant height, root length, and fresh weight of *Echinochloa crusgalli* were not significantly different after spraying water and *β*-CD, indicating that *β*-CD had no inhibition effect on the growth of *Echinochloa crusgalli*. The inhibitory effects of spraying diuron, the diuron/*β*-CD physical mixture, and the diuron–*β*-CD inclusion complex on the plant height, root length, and fresh weight of *Echinochloa crusgalli* are shown in Figure 11. The inhibitory rates of diuron on the plant height, root length, and fresh weight of *Echinochloa crusgalli* were 27%, 52%, and 56%, respectively. The inhibitory rates of the diuron/*β*-CD physical mixture on the plant height, root length, and fresh weight of *Echinochloa crusgalli* were 50%, 56%, and 50%, respectively. The inhibitory rates of the diuron–*β*-CD inclusion complex on the plant height, root length, and fresh weight of *Echinochloa crusgalli* were 61%, 60%, and 69%, respectively. Comparing the inhibitory effects of the above substances on the growth index of *Echinochloa crusgalli*, the diuron–*β*-CD inclusion complex had a stronger inhibitory effect on the growth of *Echinochloa crusgalli*, which could improve the solubility of diuron in water and increase the concentration of herbicide [39].

**Figure 12:**
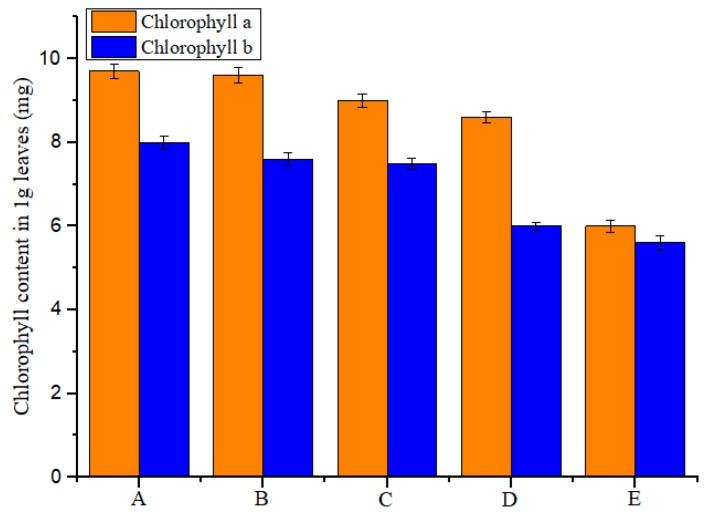
Chlorophyll content results: (**A**) water; (**B**) *β*-CD; (**C**) diuron; (**D**) physical mixture; and (**E**) the inclusion complex.

Figure 12 shows the effects of *β*-CD, diuron, the physical mixture, and the inclusion complex on the two chlorophyll contents in *Echinochloa crusgalli*. The results showed that the chlorophyll content in *Echinochloa crusgalli* was not significantly different after spraying water and *β*-CD, indicating that *β*-CD had no herbicidal activity. The chlorophyll content in *Echinochloa crusgalli* decreased after spraying diuron. The chlorophyll content of *Echinochloa crusgalli* was significantly decreased by spraying the physical mixture and the inclusion complex. The lowest chlorophyll content was obtained in *Echinochloa crusgalli* treated with the inclusion complex, indicating that the inclusion complex had strong herbicidal activity, which might be related to the ability of *β*-CD to improve the water solubility of diuron, which led to an increase in the concentration of diuron in the solution, thereby increasing the herbicidal activity.

## 4. Discussion

As one of the commonly used herbicides in agriculture, diuron has low solubility and needs to be dissolved in organic reagents during use. This will easily cause environmental pollution and threaten human safety. Therefore, it is necessary to explore a new method to improve the solubility and thermal stability of diuron in water, enhance its herbicidal ability, and reduce its environmental damage. In this study, the combination of diuron and *β*-CD to form an inclusion complex was shown to significantly change the physical properties of diuron.

The inclusion complex of diuron with *β*-CD was successfully prepared with the aid of co-precipitation. The results of FTIR, XRD, SEM TGA, DSC, and ^1^H NMR revealed that a diuron–*β*-CD inclusion complex was formed. ^1^H NMR and a diagram of the inclusion ratios were used to determine the stoichiometry of the inclusion complex. From the phase solubility study, it was found that the water solubility of diuron was improved after forming the inclusion complex. The results of the bioactivity assay showed that the diuron/*β*-CD inclusion complex presented higher herbicidal activity against *Echinochloa crusgalli*. The TGA and phase solubility studies also implied significant modifications to the thermal stability of the diuron–*β*-CD inclusion complex. In summary, the formation of the inclusion complex can improve the water solubility and thermal stability of this herbicide, improve its herbicidal ability, and reduce environmental pollution; therefore, it is worthy of further exploration and application in agriculture.

## Figures and Tables

**Table 1:** Chemical shift of inclusion complexes relative to diuron and cyclodextrin.

Compounds	Chemical Shift (δ)					
	H-1	H-2	H-3	H-4	H-6	H-5
Diuron	8.575	7.865	7.475	3.367	2.928	2.518
Inclusion complex of *β*-CD and diuron	8.551	7.845	7.461	3.335	2.921	2.507
Δδ	0.024	0.020	0.014	0.032	0.007	0.011
*β*-CD	5.750	3.347	3.653	3.300	3.629	3.603
Inclusion complex of β-CD and diuron	5.740	3.338	3.629	3.293	3.623	3.571
Δδ	0.010	0.009	0.024	0.007	0.006	0.032
